# The hnRNP-Htt axis regulates necrotic cell death induced by transcriptional repression through impaired RNA splicing

**DOI:** 10.1038/cddis.2016.101

**Published:** 2016-04-28

**Authors:** Y Mao, T Tamura, Y Yuki, D Abe, Y Tamada, S Imoto, H Tanaka, H Homma, K Tagawa, S Miyano, H Okazawa

**Affiliations:** 1 Department of Neuropathology, Medical Research Institute, Tokyo Medical and Dental University, Bunkyo-ku, Tokyo, Japan; 2 Department of Computer Science, Graduate School of Information Science and Technology, The University of Tokyo, Bunkyo-ku, Tokyo, Japan; 3 Laboratory of DNA Information Analysis, Human Genome Center, Institute of Medical Science, The University of Tokyo, Minato-ku, Tokyo, Japan; 4 Center for Brain Integration Research, Tokyo Medical and Dental University, Bunkyo-ku, Tokyo, Japan

## Abstract

In this study, we identify signaling network of necrotic cell death induced by transcriptional repression (TRIAD) by *α*-amanitin (AMA), the selective RNA polymerase II inhibitor, as a model of neurodegenerative cell death. We performed genetic screen of a knockdown (KD) fly library by measuring the ratio of transformation from pupa to larva (PL ratio) under TRIAD, and selected the cell death-promoting genes. Systems biology analysis of the positive genes mapped on protein–protein interaction databases predicted the signaling network of TRIAD and the core pathway including heterogeneous nuclear ribonucleoproteins (hnRNPs) and huntingtin (Htt). RNA sequencing revealed that AMA impaired transcription and RNA splicing of Htt, which is known as an endoplasmic reticulum (ER)-stabilizing molecule. The impairment in RNA splicing and PL ratio was rescued by overexpresion of hnRNP that had been also affected by transcriptional repression. Fly genetics with suppressor or expresser of Htt and hnRNP worsened or ameliorated the decreased PL ratio by AMA, respectively. Collectively, these results suggested involvement of RNA splicing and a regulatory role of the hnRNP-Htt axis in the process of the transcriptional repression-induced necrosis.

In CAG/polyglutamine expansion disorders including Huntington's disease and spinocerebellar ataxias, among various pathological pathways such as ubiquintin–proteasome system, autophagy, chromatin structure, synaptic function, mitochondrial function, and so on (reviewed by Orr and Zoghbi^1^and La Spada and Taylor^2^), impairment of a broad spectrum of gene expression at transcriptional and post-transcriptional levels has been implicated in the pathology because nuclear transfer of disease proteins is essential to induce large impact on phenotypes,^3, 4, 5^ mutant proteins interact with numerous transcription-related factors^6, 7, 8^ and mutant RNAs transcribed from the disease genes form nuclear speckles or cytoplasmic RNA foci including stress granules.^9, 10, 11^ The similar situation is also expected in frontotemporal lobar degeneration (FTLD) associated with RNA regulator proteins like TAR DNA-bnding protein 43 kD (TDP43) or fused in sarcoma/translocated in sarcoma (FUS),^12, 13^ suggesting that impaired expression of a wide range of genes is one of the main pathological domain in neurodegenerative diseases in general.

In neurodegenerative diseases, cell death never occurs simultaneously in a huge number of neurons in the brain. In contrast to cerebrovascular diseases, the occurrence of cell death is rather stochastic. Accumulation of cell death in the brain makes the disease phenotypes irreversible. It is enigma why the average time needed for cell death is so long, may be >10 years, in most of human neurodegenerative diseases although expression of the causative gene product is initiated from an embryonic stage.

Such slowness of cell death and the lack of definite morphological evidence in human autopsy brains have been confusing us about the nature of cell death in neurodegenerative diseases. Long discussion has been lasting on whether it is apoptosis or not. On the other hand, recent progress of necrosis^14, 15, 16^ provides another possibility for cell death of neurodegenerative diseases. Although classic necrosis was defined as a sudden cell death by strong extrinsic insults, recent studies of signaling pathways for tumor necrosis factor (TNF)-induced necrosis^17^ and necropotosis ^14, 15, 16, 18^ revealed the critical roles of RIP1/3 in necrosis,^19^ indicating that intrinsic factors could trigger necrosis. However, it is still unclear how the necrotic signaling pathway can explain the slowness of cell death in neurodegenerative diseases.

As a model connecting slow cell death and impaired trasncription, we previously proposed a unique necrosis named TRIAD (transcriptional repression-induced atypical cell death of neurons) induced by general transcriptional repression with *α*-amanitin (AMA), the RNA polymerase II (Pol II)-specific inhibitor,^20^ in mammalian neurons. TRIAD is characterized with enormous expansion of endoplasmic reticulum (ER) that is distinct from mildly enlarged ER in apoptosis.^20^

In this study, we elucidated the molecular network of TRIAD and identified huntingtin (Htt), a causative gene of Huntington's disease, and two heterogeneous nuclear ribonucleoproteins (hnRNPs) involved in splicing ^21^ as key molecules in the network. As hnRNPs like TDP43 are causative genes of neurodegeneration^22, 23, 24, 25, 26, 27, 28, 29, 30, 31, 32^ and overexpression of hnRNPs possess therapeutic effect on neurodegeneration,^33^ our data provide a critical view on ‘splicing disturbance' in neurodegenerative cell death.

## Results

### Feeding AMA induces TRIAD in fly neurons

To elucidate signaling network of TRIAD, we intended to perform genetic screen of a library of knockdown (KD) flies fed with AMA and identify modifier genes of TRIAD. Next, we also planned to draw a molecular network of TRIAD by mapping the modifier genes on protein–protein interaction (PPI) databases and to elucidate new core genes in the network, basically following the method described in our previous studies.^34, 35^

**Figure 1:**
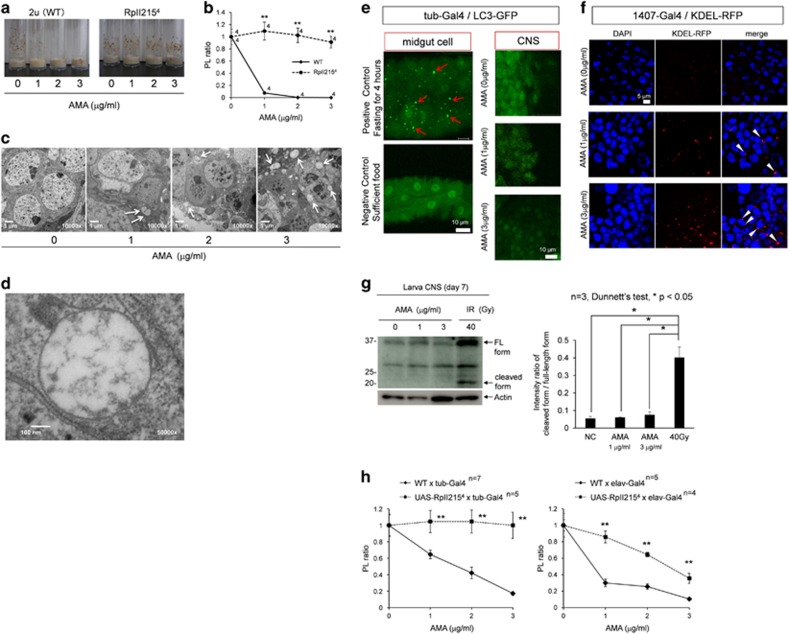
TRIAD is reproduced by AMA feeding in *Drosophila*. (**a**) In wild-type (2 u) fly, the ratio of transformation from pupa to larva (PL ratio) was decreased by AMA feeding in a dose-dependent manner (left panel). However, in the mutant fly possessing Pol II that does not interact with AMA (RpII215^4^), AMA did not decrease the PL ratio at all concentrations. (**b**) The graph shows quantitative analysis of PL ratio in 2 u and RpII215^4^ flies. Double asterisks indicate statistical difference at each AMA concentration (*P*<0.01) in Student's *t*-test. (**c**) In the CNS tissue of larva, we found that AMA-induced vacuolar changes in the cytoplasm of neurons by electron microscopy. A few vacuoles include intracellular micro-organella and seem autophagosomes (arrowheads), whereas most vacuoles do not have such structures and are similar to cytoplasmic vacuoles in TRIAD (arrows). (**d**) Representative images of TRIAD-like vacuoles at a higher magnification. (**e**) Left upper panel shows representative autophagosome (red arrow) in midgut cells in flies after fasting for 4 h as positive control. Left lower panel is the negative control. TRIAD-like vacuoles were unlikely to be autophagosomes because AMA at 1 or 3 *μ*g/ml did not increase the number or intensity of LC3-GFP-positive autophagosome. (**f**) TRIAD-like vacuoles were likely to be ER because the number and intensity of KDEL-RFP-positive ER (arrowhead) were increased by the treatment of AMA at 1 or 3 *μ*g/ml. (**g**) Western blot analysis of CNS tissues from AMA-treated larvae (day 7). The cleaved form of *Drosophila* homolog of caspase 3 (Drl ICE) was detected in CNS from irradiated larvae but not detected in the AMA-treated larvae. Right graph indicates quantitative analysis. (**h**) The relative resistance against AMA during the PL transformation by ubiquitous (left graph) and neuron-specific (right graph) expression of AMA-resistant Pol II gene by tub-Gal4 and elav-Gal4, respectively. **P < 0.01 in Student's *t*-test

Before genetic screen, we found that AMA-feeding decreased the ratio of transformation from pupa to larva (Figure 1a, left panel), and decided to use the ‘PL ratio' as a marker for the genetic screen. Simultaneously, we found that flies possessing Pol II mutations (RpII215^4^) were resistant to AMA and the mutants fed by AMA showed no change of PL ratio (Figure 1a, right panel). Quantitative analysis of wild-type (*w*^*1118*^) and RpII215^4^ flies confirmed the results (Figure 1b), supporting that PL ratio reflected AMA toxicity mediated by Pol II.^36, 37^

We also examined whether AMA-induced TRIAD in neurons of larva. Electron microscopy revealed neuronal death with extremely large cytoplasmic vacuoles in larva fed with AMA (Figure 1c). The cytoplasmic vacuoles were morphologically distinct from autophagosome at higher magnification (Figure 1d). The vacuoles were further characterized using organella markers, LC3-GFP and KDEL-RFP (Figures 1e and f). LC-GFP-positive autophagosome expressed by ubiquitous driver tub-Gal4 was not increased or enlarged by AMA (Figure 1e), whereas KDEL-RFP-positive ER driven by neuron-specific 1407-Gal4 was obviously increased and enlarged in the cytoplasm by AMA (Figure 1f) consistently with the feature of TRIAD.^20^ In addition, western blot analysis revealed that *Drosophila* homolog of caspase 3 (drl ICE) was not activated in central nervous system (CNS) tissue of AMA-fed larva (Figure 1g). These results supported that AMA-fed fly could be used as a model of TRIAD for genetic screen.

To discriminate the effects of AMA on PL ratio through neurons and non-neuronal cells, we expressed wild-type and AMA-resistant Pol II gene by tub-Gal4 or elav-Gal4, and examined the effect of AMA on PL ratio in the two sets of flies. Elav-Gal4 flies with normal Pol II were weaker for AMA than tub-Gal4 flies. However, the similar discrepancies of PL ratio between WT and AMA-resistant Pol II expresser flies indicated that the decrease of PL ratio by AMA was largely attributed to the toxicity through neurons (Figure 1h).

### Identification of the signaling network of TRIAD

**Figure 2:**
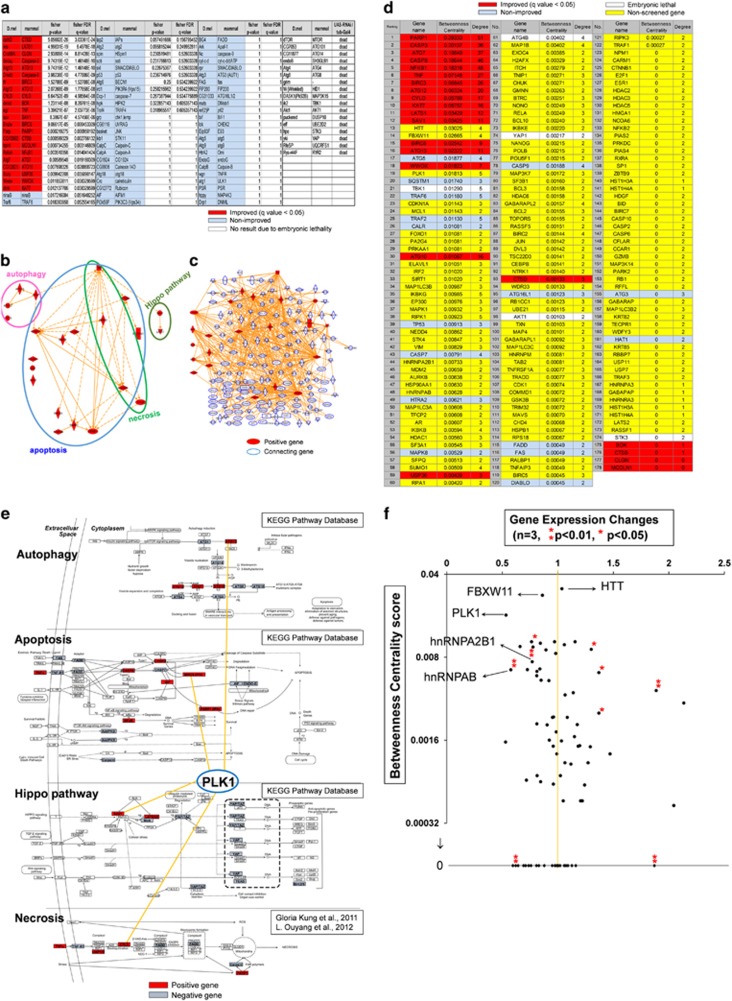
Identification of the key molecules from the signaling networks of TRIAD. (**a**) List of 93 KD flies tested in the screening whose target genes are related to apoptosis, necrosis, autophagy or hippo pathway. We tested the effect of four concentrations of AMA (0.25, 1, 2, 3 *μ*g/ml) on the PL ratio, and statistically evaluated the difference from wild-type (2 u) flies. Improvement was judged by the statistical difference (*q* value <0.05 in Fisher's FDR test). KD flies that were embryonic lethal were not colored. (**b**) Network of positive genes was generated by IPA with PPI databases including BIND, BioGrid, HPRD, IntAct and MINT. The positive genes in the network are also categorized to the groups related apoptosis, necrosis, autophagy and hippo pathway based on the database from KEGG (www.genome.jp/kegg). (**c**) The secondary network that was generated by changing the condition of networking to allow edges from two positive genes to one gene listed at least in one of the four PPI databases. (**d**) Betweenness centrality analysis of the secondary network (**c**) predicted the key molecules in TRIAD. The genes in the secondary network are listed with the scores of betweenness centrality. Positive genes in the KD fly screening are colored red, non-positive genes in the screen are colored gray and the other genes in the secondary network (not used for the KD fly screening) are colored yellow. The genes whose KD induced embryonic lethal phenotype are not colored. (**e**) Positive and negative genes in the KD fly screening are mapped on KEGG pathways. Among genes in the secondary network, only Plk1 could connect to all the pathways. (**f**) Gene expression changes in CNS tissues were analyzed by gene chip (AMA-treated/non-treated). Genes in the secondary network are plotted with the changes and the centrality scores

Therefore, we used PL ratio to screen signaling molecules of TRIAD. We prepared 93 types of KD flies whose target genes were related to apoptosis, necrosis, autophagy or hippo pathway (Figure 2a). Considering with the preliminary experiment (Figure 1c), we used four concentrations (0.25, 1, 2 and 3 *μ*g/ml) of AMA for genetic screen (Supplementary Figures 1A–D). Given that typical morphological changes were the most abundant at 3 *μ*g/ml (Figure 1d), we used the results at 3 *μ*g/ml for final judgment in the screen (Figure 2a, red-marked genes). The raw data of the screen are shown in Supplementary Figure 1 and Supplementary Table 1. To confirm that the PL ratio mainly reflected TRIAD but not apoptosis or necroptosis, we fed the fly with AMA+apoptosis inhibitor (z-VAD-fmk) or AMA+necroptosis inhibitor (necrostatin) as the negative control, respectively (Supplementary Figure 1E). As expected, these inhibitors did not change the PL ratio (Supplementary Figure 1E).

Next, we plotted the positive genes on signaling pathways of apoptosis, necrosis, autophagy and hippo supplied from Kyoto Encyclopedia of Genes and Genomes (KEGG: www.genome.jp/kegg). Interestingly they were not concentrated to a specific group (Supplementary Figure 2), indicating that the next approach to elucidate the network of TRIAD was necessary.

Following our previous methods,^34, 35^ we placed positive genes on the nodes of PPI databases including BIND (http://www.bind.ca/), BioGrid (http://www.thebiogrid.org/), HPRD (http://www.hprd.org/), IntAct (http://www.ebi.ac.uk/intact/site/index.jsf) and MINT (http://mint.bio.uniroma2.it/mint/Welcome.do). Using INGENUITY pathway analysis (IPA) (www.ingenuity.com/products/ipa), we generated a molecular network connecting positive genes by adding edges reflecting direct PPI (line: direct interaction suggested from PPI database, dot line: indirect interaction suggested from ingenuity original database based on research papers) (Figure 2b). In this case, we found some interactions beyond functional gene groups (Figure 2b). Furthermore, we changed the condition of networking and allowed edges from two positive genes to one gene unselected by Fly screen but listed in PPI databases (Figure 2c). These networks were considered as prototypes of the TRIAD signaling network.

### Identification of the key molecules in the network of TRIAD

To predict critical factors in the TRIAD signaling network, we performed betweenness centrality analysis. First, we calculated the centrality score of each node in the network and ranked the gene according from the highest score. Naturally positive genes were ranked at high positions (Figure 2d) because they were used as hubs in the network for betweenness centrality analysis. Interestingly, three genes that had not been tested in our screen were ranked at high positions equivalently to the positive genes from the screen (Figure 2d). They were Htt, F-Box and WD repeat domain containing 11 (FBXW11) and polo-like kinase-1 (Plk1). Htt- and FBXW11-KD flies were not included in our list of cell death-related genes and not tested in our genetic screen. Thus, further investigation was needed to evaluate their roles in TRIAD.

Among these genes listed at high position, only Plk1 was connected to all the functional groups of apoptosis, necrosis, autophagy and hippo pathway, supporting the central role of Plk1 in TRIAD (Figure 2e). Meanwhile, *Drosophila* homolog of yes-associated protein (YAP), yorkie (yki), was again included in the list of high centrality genes (Figure 2d, at 74 position) although YAP/yki KD fly was lethal in our fly genetic screen, and therefore it had not been included as a positive gene for networking. Instead of the neglect in the genetic screen, the following centrality analysis successfully recaptured the significance of YAP in TRIAD signaling network.

We further analyzed relationship of the centrality score and the transcriptional change in the CNS of AMA-fed pupa (3 *μ*g/ml) (Figure 2f). Despite of their high centrality scores, Htt, FBXW11 and Plk1 were not changed in their expression at gene chip level (Figure 2f). On the other hand, some of the genes whose expression levels were significantly changed show a relatively high centrality scores (Figure 2f). These genes including hnRNPA2B1 and hnRNPAB were considered candidate key molecules in the TRIAD signaling cascade (Figure 2f).

### Secondary impairment of RNA splicing by transcriptional repression in fly

**Figure 3:**
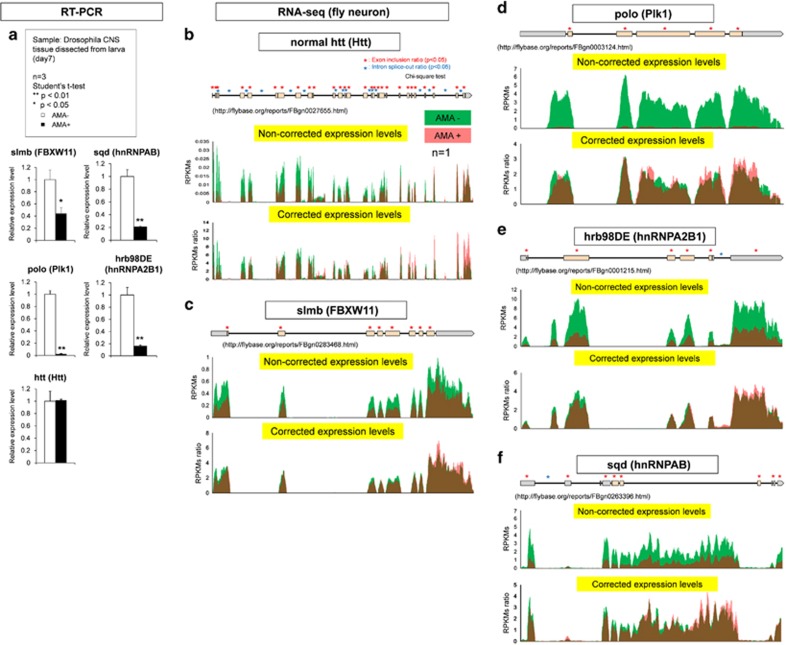
Impaired RNA splicing in TRIAD of fly CNS. (**a**) Transcriptional changes of candidate key genes in the CNS tissues by AMA (3 *μ*g/ml) were evaluated by RT-PCR. (**b**) Transcriptional levels and splicing changes of fly Htt by AMA were evaluated by RNA sequencing with a NGS. Non-corrected expression levels represent total expression levels with RPKMs. Corrected expression levels represent RPKM ratio corrected by total gene expression levels of Htt in the absence or presence of AMA. (**c**) Transcriptional levels and splicing changes of fly FBXW11 (slmb) were evaluated by RNA sequencing with a NGS. (**d**) Transcriptional levels and splicing changes of fly Plk1 (polo) were evaluated by RNA sequencing with a NGS. (**e**) Transcriptional levels and splicing changes of fly hnRNPA2B1 (hrb98DE) were evaluated by RNA sequencing with a NGS. (**f**) Transcriptional levels and splicing changes of fly hnRNPAB (sqd) were evaluated by RNA sequencing with a NGS

Before examination of functions of such candidate key molecules in TRIAD, we tested whether their expression levels were actually suppressed by AMA at mRNA level. As expected, qPCR confirmed the reduction of slmb (*Drosophila* homolog of FBXW11) and polo (*Drosophila* homolog of Plk1) (Figure 3a). Unexpectedly, however, we could not confirm the reduction of Htt mRNA not only in the brain tissue but also in the whole body of *Drosophila*, when we used the primers at the C-terminal region for RT-qPCR (Figure 3a).

To find reasons for the discrepancy, we performed RNA sequencing with CNS tissues of pupa. Consequently we found that AMA markedly changed splicing pattern of Htt (Figure 3b). When the expression levels were normalized by average RPKMs, exon inclusion and intron splice-out were changed in most exons and introns (Figure 3b). Such splicing change of Htt could be the reason for the discrepancy. RNA sequencing also revealed that AMA affected splicing patterns of the other key genes like FBXW11 and Plk1 (Figures 3c and d).

Furthermore, we revisited gene list of the TRIAD network (Figure 2d) to search candidates that affect RNA splicing, and found that *Drosophila* hnRNPAB/sqd and hnRNPA2B1/hrb98DE. We suspected that transcriptional repression of hnRNPs may influence the splicing pattern of Htt. Actually qPCR revealed that AMA decreased hnRNPAB and hnRNPA2B1 mRNAs (Figure 3a). Interestingly, RNA sequencing revealed that not only transcription but also RNA splicing were changed in these splicing-related molecules^38, 39, 40^ (Figures 3e and f).

### Secondary impairment of RNA splicing by transcriptional repression in mammalian neuron

To test whether the secondary impairment of RNA splicing by transcriptional repression of hnRNPs is shared by mammalian neurons, we added AMA to the primary culture of rat cortical neurons, and examined splicing patterns by RNA sequencing. As expected, RNA splicing was impaired. The changes were especially remarkable in Htt, FBXW11 and Plk1 (Supplementary Figures 2A–C). On the other hand, the splicing changes were relatively small in hnRNPA2B1 and hnRNPAB, whereas their total expression levels were markedly changed (Supplementary Figures 2D and E).

**Figure 4:**
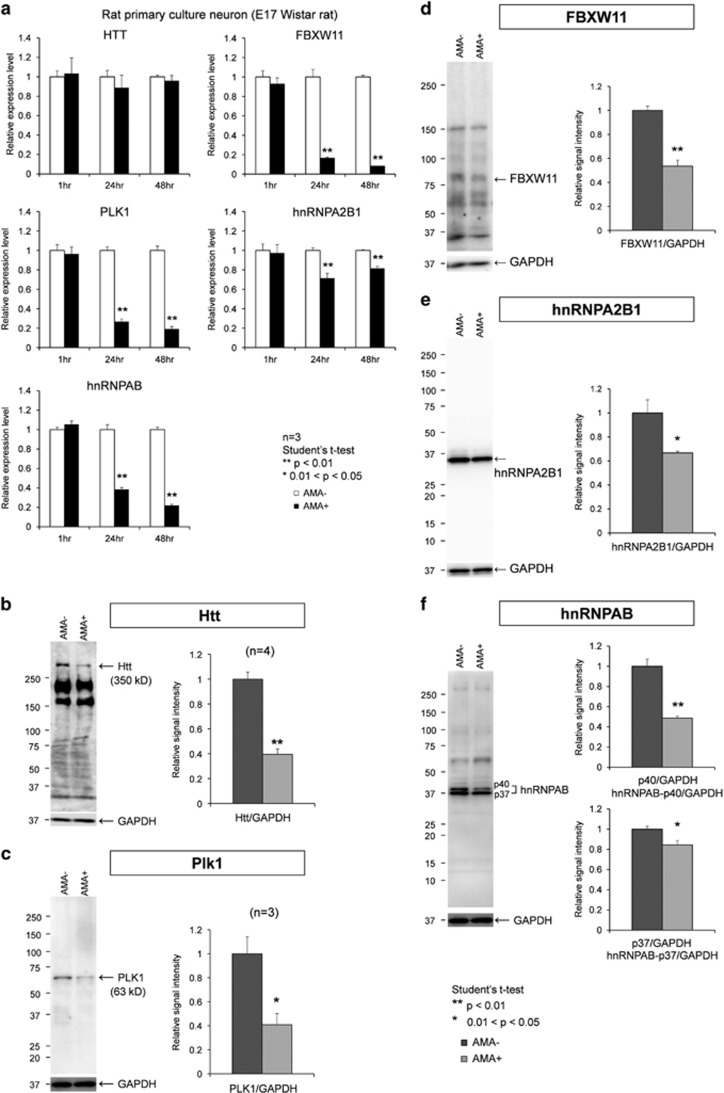
Chronological changes of RNA and protein expression levels in rat cortical neurons during TRIAD. (**a**) Chronological changes of RNA expression levels of candidate key genes were evaluated by RT-qPCR from 1 to 47 h after addition of AMA to primary culture of rat cortical neurons. (**b**) Protein expression levels of rat Htt were evaluated by western blot before and 24 h after addition of AMA to primary culture of rat cortical neurons. Right graph shows quantitative analysis. (**c**) Protein expression levels of rat Plk1 were evaluated by western blot before and 24 h after addition of AMA to primary culture of rat cortical neurons. Right graph shows quantitative analysis. (**d**) Protein expression levels of rat FBXW11 were evaluated by western blot before and 24 h after addition of AMA to primary culture of rat cortical neurons. Right graph shows quantitative analysis. (**e**) Protein expression levels of rat hnRNPA2B1 were evaluated by western blot before and 24 h after addition of AMA to primary culture of rat cortical neurons. Right graph shows quantitative analysis. (**f**) Protein expression levels of rat hnRNPAB were evaluated by western blot before and 24 h after addition of AMA to primary culture of rat cortical neurons. Right graphs show quantitative analyses of two isoforms

All these changes were basically confirmed by RT-qPCR, and the chronological observation revealed that only Htt did not show a remarkable change in transcription at RT-qPCR level (Figure 4a). However, as expected from remarkable changes in splicing (Supplementary Figure 2A), protein expression levels were markedly changed in these candidate key molecules for TRIAD including Htt, FBXW11, Plk1, hnRNPA2B1 and hnRNPAB (Figures 4b–e).

### Deficiency of hnRNP causes secondary impairment of RNA splicing

**Figure 5:**
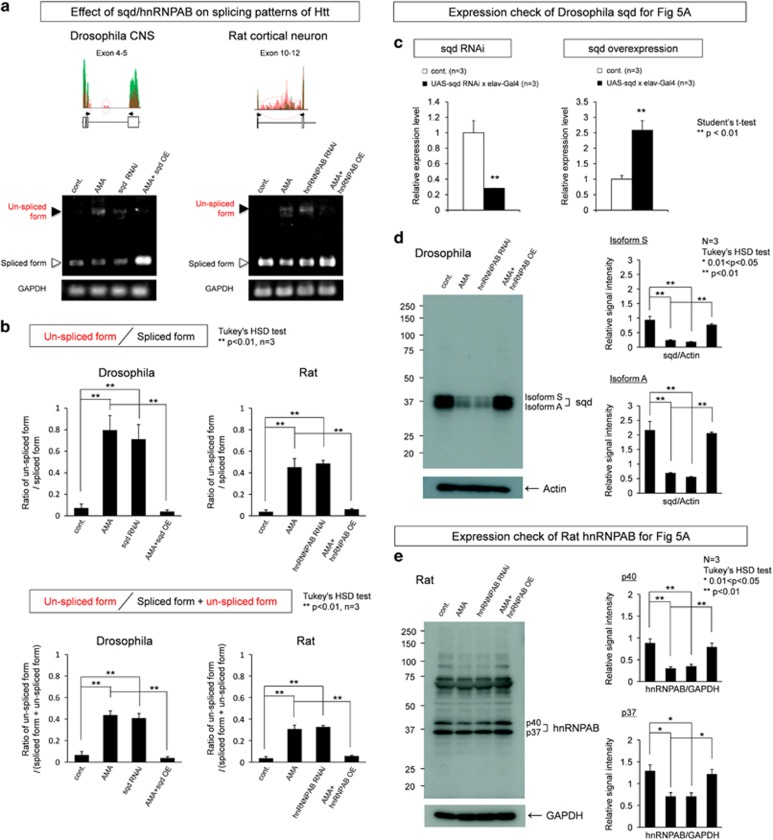
Genetic impairment and rescue of splicing by hnRNPAB. (**a**) (Left panel) KD of hnRNPAB in fly (UAS-sqd-RNAi x elav-Gal4) reproduced the spicing impairment of *Drosphila* htt RNA in AMA (3 *μ*g/ml)-fed flies, whereas overexpression of hnRNPAB rescued the impairment. The upper band is the PCR product from unspliced pre-mRNA. (Right panel) KD of hnRNPAB reproduced in rat primary cortical neurons reproduced the spicing impairment of rat Htt in AMA-treated rat primary cortical neurons, whereas overexpression of hnRNPAB rescued the impairment. The upper band is the PCR product from unspliced pre-mRNA. (**b**) The ratio between spliced and unspliced RNA of *Drosophila* and rat Htt was analyzed in two parameters. (**c**) RT-qPCR was performed to confirm KD (UAS-sqd-RNAi x elav-Gal4) and overexpression of sqd (UAS-sqd-RNAi × elav-Gal4) in flies used for (**a**) – left panel. CNS was dissected and total RNA was extracted for RT-pPCR. (**d**) Western blot analysis was performed to confirm KD (UAS-sqd-RNAi × elav-Gal4) and overexpression of sqd (UAS-sqd-RNAi x elav-Gal4) in flies used for (**a**) – left panel. CNS was dissected for WB. (**e**) Western blot analysis was performed to confirm KD and overexpression of hnRNPAB in rat primary cortical neurons used for (**a**) – right panel

To test our hypothesis that transcriptional decrease of hnRNPs causes the secondary splicing impairment of target genes, we performed RT-PCR of Htt, the representative gene markedly changed in splicing by AMA, and examined whether down- or upregulation of hnRNP affects the splicing pattern of the possible target gene (Figure 5a).

*Drosophila* homolog of mammalian hnRNPAB is sqd. Central nervous tissues of wild-type (*w*^*1118*^), AMA-treated wild-type, sqd-KD fly (UAS-sqd-RNAi/elav-Gal4) and AMA-treated sqd-OE fly were forwarded to RT-PCR to check splicing pattern of fly htt. Non-spliced RNA was increased by AMA and by sqd-KD, whereas sqd overexpression rescued the impaired splicing by AMA (Figures 5a and b). RT-qPCR and western blot analysis confirmed that expression level of sqd was actually decreased or increased in CNS of KD or overexpression (OE) flies, respectively (Figures 5c and d).

We also performed similar experiments with rat primary cortical neurons. We used hnRNPAB-siRNA and pCMV-hnRNPAB to KD and overexpress hnRNPAB, respectively. Their effects on the splicing of Htt pre-mRNA in rat primary cortical neurons were similar to the case in fly (Figures 5a–d). KD of hnRNPAB impaired RNA splicing just like AMA did, and the impairment of RNA splicing by AMA was rescued by OE of hnRNPAB. The expression levels of hnRNPAB in primary neurons after KD and OE were confirmed by western blot analysis (Figure 5e). These results strongly suggested that AMA impairs RNA splicing of Htt through the transcriptional repression of hnRNPAB both in rat and *Drosophila*.

### Rescue of TRIAD-dependent PL ratio by Htt, hnRNP and YAP

**Figure 6:**
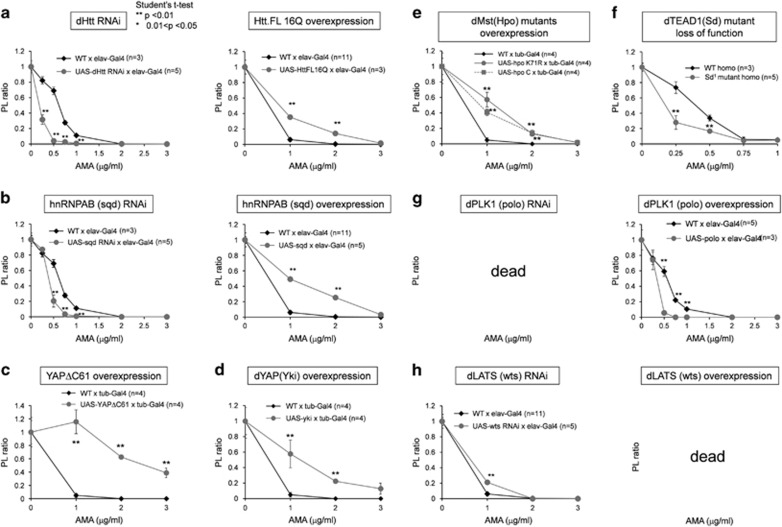
Genetic exacerbation and rescue of fly TRIAD by key genes. (**a**) Exacerbation and rescue of the PL ratio of AMA-fed flies by fly Htt KD and human Htt16Q overexpression. (**b**) Exacerbation and rescue of the PL ratio of AMA-fed flies by fly hnRNPAB (sqd) KD and overexpression. (**c**) Rescue of the PL ratio of AMA-fed flies by overexpression of rat YAPΔC61. (**d**) Rescue of the PL ratio of AMA-fed flies by overexpression of fly YAP homolog, Yki. (**e**) Rescue of the PL ratio of AMA-fed flies by overexpression of dominant-negative mutant of fly MST homolog, Hpo. (**f**) Exacerbation of the PL ratio of AMA-fed flies by overexpression of fly WW45 homolog, Salvador (Sd) mutant. (**g**) Overexpression of fly Plk1 homolog, polo, exacerbated the PL ratio of AMA-fed flies. On the other hand, KD of polo induced lethal phenotype probably because polo deficiency causes the impairment of cell proliferation during development. (**h**) KD of fly Lats homolog, Wts, weakly rescued the PL ratio of AMA-fed flies, whereas overexpression of Wts induced embryonic lethal phenotype

Further to examine the significance of the hnRNP-Htt axis in TRIAD, we examined KD and OE of normal Htt and hnRNPAB on PL ratio. When the two molecules were knocked down, the decrease of PL ratio was accelerated (Figures 6a and b). On the other hand, when the two molecules were overexpressed, the decrease of PL ratio was recovered (Figures 6a and b). These results supported the critical role of the hnRNP-Htt axis in TRIAD.

To re-examine our previous result regarding the role of YAP in TRIAD,^20^ we also examined the effect of YAPΔC or YAP/yki overexpression on PL ratio. In consistence with our previous result that YAPΔC functions as a protector against TRIAD just like full-length YAP,^20^ OE of YAPΔC and yki rescued the PL ratio (Figures 6c and d). We found that YAP1 was ranked at 74th place in centrality analysis (Figure 2d) and YAP KD made the pupa lethal (Figure 2a). Therefore, this study also supported that YAP is another domain in signaling network of TRIAD.

We extended the genetic analysis to other molecules related to Hippo pathway.^41^ First, two types of dominant-negative mutants of Hippo/Mats both improved the PL ratio (Figure 6e). Second, hypomorph mutant of *Drosophila* homolog of TEAD (scalloped, Sd) worsened the PL ratio (Figure 6f). In this experiment, the lower concentrations of AMA than the other graph were used to detect the worsening by Sd mutation (Figure 6f). KD of PLK1/polo induced lethal phenotype at pupa stage (Figure 6g), whereas OE of polo improved the PL ratio slightly (Figure 6g). Suppression of wts, *Drosophila* homolog of LATS, improved the PL ratio slightly (Figure 6h), whereas OE of wts was lethal (Figure 6h).

**Figure 7:**
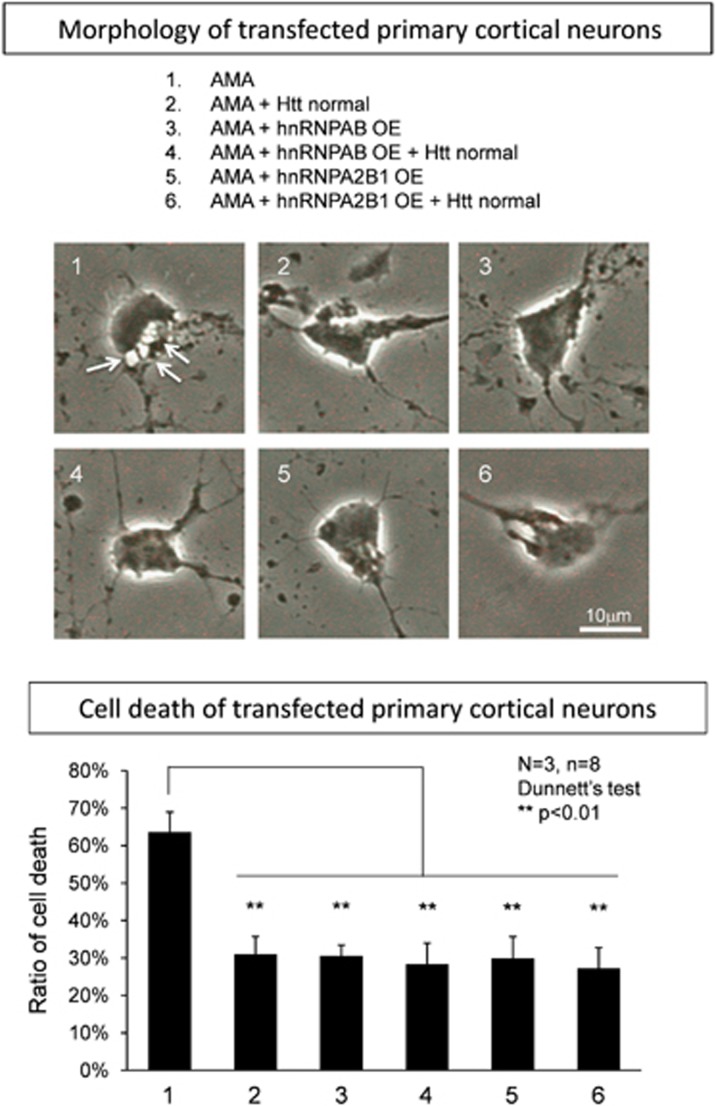
Normal Htt and hnRNAP suppress TRIAD of primary neurons. The effect of overexpressing normal Htt, hnRNPAB and hnRNPA2B1 on TRAID was investigated. All the three genes suppressed AMA-induced cell death of primary rat cortical neurons, whereas additive effect between Htt and hnRNP was not observed. Twenty-four hours after transfection of the expression vectors to primary rat cortical neurons, AMA was added to the medium at a final concentration of 25 *μ*g/ml, and the ratio of cell death was evaluated by Trypan blue dye exclusion after another 3 days

Moreover, to confirm the significance of hnRNP-Htt axis in mammalian neurons, we tested whether OE of hnRNPs and/or normal Htt could inhibit TRIAD in rat primary cortical neurons (Figure 7). As expected, hnRNPAB, hnRNPA2B1 and normal Htt suppressed TRIAD (Figure 7). Additive effect of the hnRNP-normal Htt co-expression on TRIAD was not observed, supporting that they are directly linked as the upstream and downstream molecules in the same pathway.

### Pharmacological rescues of TRIAD-dependent PL ratio and rat primary neuron TRIAD by Plk1 inhibitors

**Figure 8:**
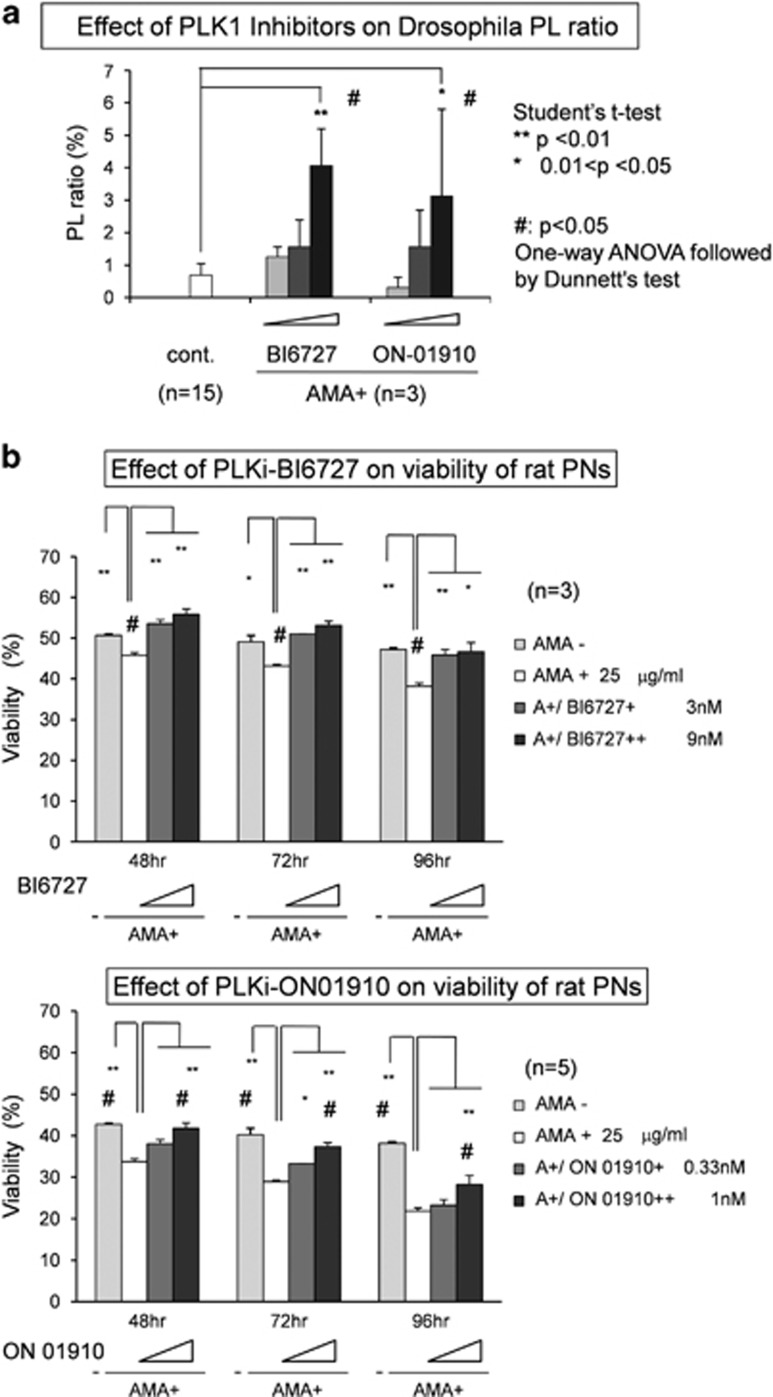
Chemical rescue of fly and rat TRIAD by Plk1 inhibitors. (**a**) The effects of two Plk1 inhibitors on the PL ratio of AMA-fed flies. * and **P<0.01 and *P*<0.05, Student's *t*-test. ^#^*P*<0.05, One-way ANOVA followed by Dunnett's test. (**b**) The effects of two Plk1 inhibitors on the viability of primary neurons under the AMA-induced cell death in primary culture of rat cortical neurons. * and **P<0.01 and *P*<0.05, Student's *t*-test. ^#^*P*<0.05, One-way ANOVA followed by Dunnett's test

Finally, we tested the effect of two specific kinase inhibitors against Plk1, BI6727 and ON01910, on TRIAD. The therapeutic effect of BI6727 and ON01910 on the PL ratio was obvious when pupae were fed with AMA with BI6727 or ON01910 (Figure 8a). The effect of BI6727 and ON01910 on TRIAD of rat primary cortical neurons (E17) was directly evaluated by their viability after AMA treatment. At 48, 72 and 96 h after addition of AMA, viability of neurons were evaluated with Trypan blue assay.^20^ The results obviously indicated the protective effect of these Plk1 inhibitors against TRIAD (Figure 8b). These results with BI6727 and ON01910 may be applicable to the development of pharmaceuticals against TRIAD or TRIAD-like necrosis that could occur in neurodegenerative diseases.

## Discussion

### Involvement of splicing in TRIAD

In this study, we intended to elucidate molecular network of TRIAD taking advantage of a library of KD flies and bioinformatics based on PPI network database.^42^ Through screening of KD fly library, we identified modifier genes of TRIAD. Mapping them on the high-quality PPI databases, we identified the molecular network of TRIAD crossing over multiple signaling pathways.

**Figure 9:**
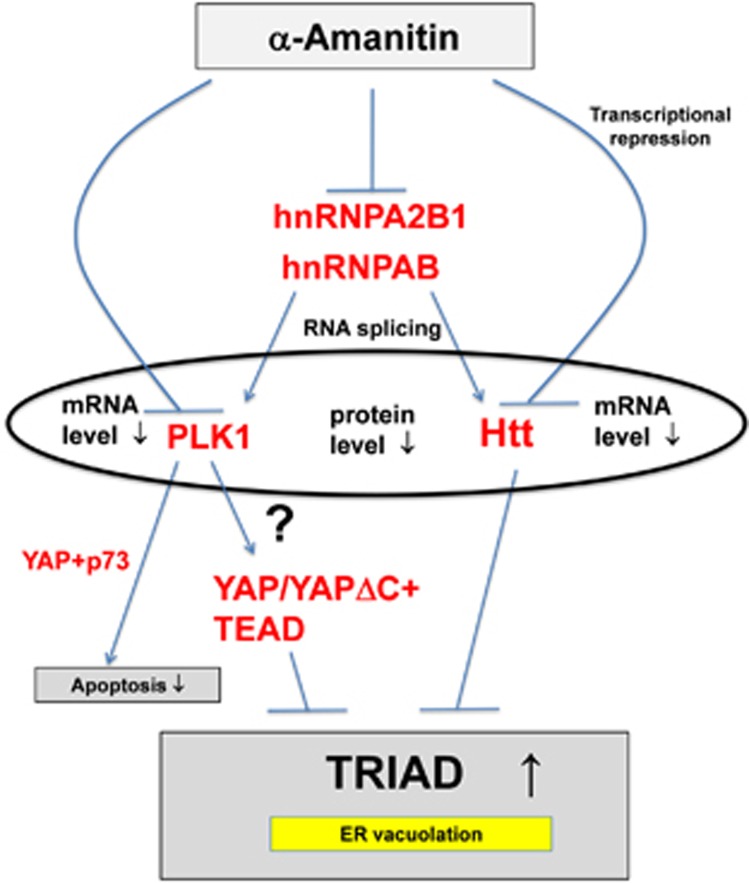
Signaling pathways of TRIAD. Hypothetical signaling pathways of TRIAD expected from the results in this study. Arrows indicate the effect of AMA. Question mark indicates the pathway without sufficient supporting results in this study

In addition, our systems biology analyses elucidated that hnRNPs and Htt, the causative gene product of Huntington's disease, may have critical roles in the network (Figure 9). The role of Htt was linked to the ER shape, as discussed below. Another intriguing finding in this study was that RNA splicing-related molecules, hnRNPs, were decreased in TRIAD both of fly and rat, leading to the splicing impairment of Htt. We therefore proposed the axis from hnRNPs to Htt mediated by the change of RNA splicing could be a new domain of TRIAD.

### hnRNPs and RNA splicing

Pol II generates many transcripts from a gene while only a part of transcripts maturate to mRNA.^21^ The premature heterogenous RNA forms large complexes with proteins called hnRNPs, which regulate multiple functions during and after RNA maturation including modification, splicing, export, localization, translation and stability.^43^ RNA splicing is mediated by *cis*-elements existing in heterogenous nuclear RNA (hnRNA) called exonic splicing enhancer/silencer (ESE/ESS) and intronic splicing enhancer/silencer (ISE/ISS). ESE and ISE are shown regulated mainly by SR proteins, whereas ESS and ISS are suspected regulated by hnRNPs.^44, 45, 46, 47, 48^ Therefore, it is quite plausible that the reduction of hnRNPA2B1 and hnRNPAB to <20% by AMA affects RNA splicing of a number of genes.

### hnRNPs in neurodegeneration

A large number of recent reports suggested that hnRNPs have critical roles in various neurodegenerative diseases and developmental disorders. For instance, mutations of hnRNPA1 were reported with ALS patients,^22^ the aggregate protein in neurons of FTLD (TDP43) is an hnRNP by itself and interacts with hnRNPA2,^49^ hnRNPA2B1 was implicated in Fragile X-associated tremor/ataxia syndrome (FXTAS) and shown to bind with riboCGG repeat,^50^ TDP43 compensate the impairment by loss of hnRNPA2B1 in fly model,^33^ hnRNPA3 binds to GGGGCC repeats in FTLD-ALS linked to C9orf72,^51^ selective deficiency of hnRNP in entorhinal cortices of AD patients and the model were reported,^52^ AUUCU expansion of Ataxin-10 impairs the functon of hnRNPK,^53^ and so on. Polyglutamine binding protein-1 (PQBP1), an RNA binding protein that forms nuclear speckles and has a low complexity domain/intrinsically denatured domain, is also an hnRNP.^54^ In other neurological diseases, antibodies against hnRNPA1 were detected in HAM/TSP patients.^55, 56^

All these results have shown a generally shared pathological significance of hnRNPs in multiple neurodegenerative diseases. Our results in this study have linked the impairment of hnRNPs to a necrotic cell death, TRIAD.

### Functional loss of Htt links to the morphological feature of TRIAD

Functional role of Htt in the maintenance of ER has been already reported.^57^ Htt and the other molecules like CLIMP63 bridge ER and microtubules to stabilize the morphology off ER.^57^ Morphological change by siRNA-mediated KD of Htt was already reported.^58^ The experiment revealed fusion of vesicles in ER-Golgi network,^58^ which is basically consistent with our observation with TRIAD in this study.

The loss of physiological function of Htt may unify neuronal death in HD and TRIAD as mutant Htt impairs function of normal Htt.^59^ The relationship between two forms of necrosis should be further investigated.

### Dual signaling pathways in neurodegenerative diseases

This study indicated that the dual pathways, direct as well as indirect impairments of gene expression of key genes, contribute to TRIAD. In the direct pathway, expression of key genes was decreased by transcriptional impairment by Pol II inhibition. In the indirect pathway, RNA splicing of key genes was impaired through the decrease of hnRNP by transcriptional repression.

Multiple diseases proteins involving Atxn1, Atxn2, Atxn7, TDP43 and FUS are related to RNA splicing.^60, 61^ A target protein of multiple neurodegenerative disease proteins, PQBP1, is deeply involved in RNA splicing, transcription and translation.^54, 62, 63^ These previous results are basically consistent with the idea that gene expression is impaired at multiple levels.

### The slow cell death

As temporal dynamics of signaling becomes slower in a complex network including multiple branches and feedback loops rather than in a simple cascade,^64^ the complexity of TRIAD signaling network (Figure 2) may be a cause why the cell death process is so slow in neurodegeneration.

Our study is not complete. We used *Drosophila* CNS instead of human neuron to identify the signaling network. Information of temporal dynamics of nodes and edges was not clarified. Signaling could be specific in each single neuron. However, our results would make sense as a preliminary approach at this time point and actually cast a light on the TRIAD network, a candidate model of neurodegeneration.

## Materials and Methods

### Fly stocks and rearing conditions

All flies culture and mating were carried at 25 °C and 60% humidity under a 12 : 12-h light–dark cycle. The AMA-resistant Pol II fly, v^1^RpII215^4^ (107164) were obtained from Drosophila Genetic Resource Center (DGRC, Kyoto, Japan). The AMA-resistant Pol II overexpression fly, pUAST-RpII215^4^, and its control w^1118^ were obtained from BestGene Inc. (Chino Hills, CA, USA). All 89 UAS-RNAi lines and corresponding control flies were obtained from the Vienna Drosophila RNAi Center (VDRC, Vienna, Austria) or the National Institute of Genetics (NIG-Fly, Shizuoka, Japan). The tublin-Gal4/TM3 GFP, Ser fly was made by crossing w;+/+sb/ TM3 GFP, Ser with tublin-Gal4/ sb^1^. The w;+/+sb/TM3 GFP, Ser fly was a gift from Dr. Sone (Toho University). The tublin-Gal4/ sb^1^ and the +/+ elav-Gal4/elav-Gal4; +/+ flies were obtained from Bloomington Drosophila Stock Center (BDSC 5138, 8765). Except BestGene and the RNAi flies, all flies were backcrossed with our wild-type flies w(CS10) ^65^ for six times before use.

### AMA-resistant test

Fifteen subjected virgin female flies were crossed with five males of tublin-Gal4/TM3.GFP.Ser and elav-Gal4 (II) on the instant food including different concentration of AMA (0, 0.25, 0.5, 0.75, 1, 2 and 3 *μ*g/ml; AMA, Calbiochem, San Diego, CA, USA; cat. #129741), respectively. After 7 days, the parents were discarded, then after 3 days, the number of the pupa was recorded.

### Electron microscopy imaging

CNS was dissected from a third instar larva in 1xPBS, fixed in 2.5% glutaraldehyde/2% formaldehyde/0.1 M phosphate buffer (pH 7.4) for 2 h at 4 °C, and washed with 0.1 M phosphate buffer for five times of 10 min at 4 °C. Then it was treated with 1% OsO_4_/0.1 M phosphate buffer for 1 h at 4 °C. The fixed CNSs were dehydrated through a graded ethanol series and embedded in epoxy resin. Ultrathin sections were stained with uranyl acetate and lead citrate. Data acquisition was performed with a transmission electron microscope (H-9000; Hitachi, Tokyo, Japan) at 24 °C.

### Confocal microscopy imaging

CNS was dissected from a third instar larva of tublin-Gal4/LC3-GFP and 1407/KDEL-RFP in 1xPBS and mounted with vectashield with DAPI (Vector Laboratories Inc., Burlingame, CA, USA), and directly observed with a confocal microscope (LSM510META; Carl Zeiss, Oberkochen, Germany). Data acquisition was performed with the Zeiss software (ZEN2009).

### Western blot analyses of fly

Flies were mated in the food without or with AMA (1 *μ*g/ml and 3 *μ*g/ml), and 100 CNSs were dissected and dissolved in 80 *μ*l 2 × sample buffer (62.5 mM Tris-HCl, pH 6.8, 2% SDS, 2.5% 2-mercaptoethanol, 5% glycerin and 0.0025% bromophenol blue). Positive control of Figure 1g was prepared by treating living second instar larvae (AMA non-treated) with X-ray (40 Gy). Primary and secondary antibodies were diluted as follows: *Drosophila* ICE (drICE, caspase-3) antibody (Cell Signaling Technology, Danvers, MA, USA; cat. #13085) at 1 : 2000. ECL peroxidase-labeled anti-rabbit antibody (GE Healthcare, Buckinghamshire, UK; cat. #NA934VS) at 1 : 10 000. *Drosophila* sqd antibody (DSHB, Sqd A 2G9) at 1 : 1000, ECLTM peroxidase-labeled anti-mouse antibody (GE Healthcare; cat. #NA931VS) at 1 : 10 000. Actin antibody (Merk Millipore, Darmstadt, Germany; cat. #MAB1501R) at 1 : 5000. ECL peroxidase-labeled anti-mouse antibody (GE Healthcare; cat. #NA931VS) at 1 : 10 000.

### Systems biology analyses

Systems biology analyses were performed using Ingenuity-IPA software (Ingenuity Systems, Inc., Redwood City, CA, USA), as described previously ^34^ based on human databases. We used two different methods for path-explorer analysis. In direct connection analysis, we used the eight popular PPI databases (BIND, BIOGRID, Cognia, DIP, INTACT, Interactome studies, MINT and MIPS) and ingenuity original database including ‘indirect interactions' based on research papers (miRecords, TarBase, TargetScan Human, Clinical Trials.gov, Gene Ontology, GVK Biosciences, KEGG, miRBase, MGD, Obesity Gene Map Database). As drawing shortest+1 network with all databases was beyond the IPA capacity, we used the four most popular PPI databases (BIND, BIOGRID, INTACT and MINT) in shortest+1 analysis. Enrichment analysis was performed by Fisher's exact test with the B-H multiple test correction to calculate q-values.

### Betweenness centrality

We used the betweenness centrality as the score of the node. The betweenness centrality of a node in a network is defined as the ratio of the node appearing in all the shortest paths between any two nodes in the network.^66^ A node with high betweenness centrality can be interpreted as one that is connected with many nodes in terms of the information flow of paths. Therefore, it may affect many biological pathways and have important roles in these pathways.^67^

### Quantitative RT-PCR

Total RNA was purified with RNeasy mini kit (Qiagen, Limburg, The Netherlands) protocol. To eliminate genomic DNA contamination, on-column DNA digestion was carried out for each sample with DNase I (Qiagen). The purified total RNA was reverse-transcribed with SuperScript VILO (Invitrogen, Carlsbad, CA, USA). Quantitative PCR analyses were performed with the 7500 Real-Time PCR System (Applied Biosystems, Foster City, CA, USA) using the Thunderbird SYBER Green (TOYOBO, Osaka, Japan). The primer sequences were:

rat_HTT, forward primer: 5′-GAGTCTAGGATGGCACTGTGGAG-3′ and reverse primer: 5′-GCTGATGAAAGCAAGGTGAGAA-3′

fly_htt, forward primer: 5′-AAGGACGCTAGTGGGCATTAG-3′ and reverse primer: 5′-AGTACAAAGCGTGGGGCTTG-3′

rat_FBXW11, forward primer: 5′-TTGTGTTCTTGGTGACGTGCTG-3′ and reverse primer: 5′-TGACTGGCGGTCTAAGGAGAAG-3′

fly_slmb, forward primer: 5′-GGCCTTGATGGAACATTAACATTTTC-3′ and reverse primer: 5′-ATCGTCGTCCTCCTCTCCTTC-3′

rat_PLK1, forward primer: 5′-CCATGGTAGACAAGCTGCTG-3′ and reverse primer: 5′-AGGCCCAGAGCTAGCAGAG-3′

fly_polo, forward primer: 5′-CTGGGCAATCTCTTCCTCAAC-3′ and reverse primer: 5′-TTCGCTCGCCCTCATACTC-3′

rat_hnRNPA2B1, forward primer: 5′-AACGTGCTGTGGCAAGAGAG-3′ and reverse primer: 5′-CTTAATTCCACCAACGAACAG-3′

fly_hrb98DE, forward primer: 5′-GCAACTATGGCGGAAACAATC-3′ and reverse primer: 5′-TGAGCGACTTTTACTTTATCACCTC-3′

rat_hnRNPAB, forward primer: 5′-ATAACTACAAGCCATACTGAGAGGC-3′ and reverse primer: 5′-GACAGGCCATAAAAGTTTGCCAAG-3′

fly_sqd, forward primer: 5′-CACGTCGAGTAATTACCATCAAAAC-3′ and reverse primer: 5′-CTCTATGCGGCTGAGGCTCTACTTAG-3′

rat_GAPDH, forward primer: 5′-AGCCCAGAACATCATCCCTG-3′ and reverse primer: 5′-CACCACCTTCTTGATGTCATC-3′

fly_gapdh1, forward primer: 5′-GGTACGACAACGAGTTCGGTTACTC-3′ and reverse primer: 5′-TTTTGGCTAGTTTAGTCCTTGCTCTG-3′.

### RNA-sequencing

AMA-non-treated or treated central neuron systems of wild-type *Drosophila* larvae as well as neuron cells of E17 Wistar rat embryos were collected and homogenized in 350 *μ*l RNA RLT buffer (Qiagen)/0.01% 2-mercaptoethanol (Wako, Tokyo, Japan), respectively. Total RNA was purified with RNeasy mini kit (Qiagen). To eliminate genomic DNA contamination, on-column DNA digestion was carried out for each sample with DNase I (Qiagen). Prepared RNA samples were subjected to a HiSEq based RNA-seq by TAKARA (700 million-bp reads). Gene expression of each sample were calculated using IGV Tools (IGV version 2.3.55, Broad Institute, Cambridge, MA, USA), and differential expression of the genes were analyzed with R (version 3.1.3, R Foundation for Statistical Computing, Vienna, Austria). Gene expression was calculated as reads per kilobase of exon per million mapped reads (RPKMs).

### Analysis of splicing change

For each gene, expression values of exons and introns were calculated as RPKMs and normalized using their average RPKMs. To detect splicing changes of exon between non-treated and AMA-treated groups, the ratio of expression values of exon and gene were compared. In all, 2 × 2 contingency table was constructed using RPKMs of exon and its gene expression, and chi-square test was used for judging statistical significance. For the change of intron splice-out, 2 × 3 contingency table was constructed using RPKMs of intron and its neighboring two exons. *P*-values were adjusted by Bonferroni method for each gene. Splicing changes were judged significant at *P*<0.05. For computing chi-square statistics appropriately, each cell in contingency table had to contain >10.

### Primary neuron culture

Primary neuron cultures were performed by same method in previous paper.^20^ In short, cerebral cortex tissues from E17 Wistar rat were treated with 0.25% trypsin/PBS at 37 °C for 15 min with gentle shaking. After following DNase I treatment, tissues were dissociated by gently pipetting with blue tips. The dissociated cells were then filtered by nylon mesh (pore size of 70 *μ*m) and were collected and plated on six-well dishes coated by poly-l-lysine (Sigma-Aldrich, St. Louis, MO, USA) at 16.3 × 10^5^ cells per well. Twenty-four hours after plating, AMA was added to the medium at a final concentration of 25 *μ*g/ml.

### Western blot analyses of E17 rat primary culture neuron

In all, 16.3 × 10^5^ cells per well from six-well dish were dissolved in 80 *μ*l of 2 × sample buffer (62.5 mM Tris-HCl, pH 6.8, 2% SDS, 2.5% 2-mercaptoethanol, 5% glycerin and 0.0025% bromophenol blue). Primary and secondary antibodies were diluted as follows: rabbit anti-Htt, 1 : 100 000 (HD1, a gift from Professor Erich E Wanker;^68^ mouse anti-Plk1, 1 : 500 (Invitrogen, Waltham, MA, USA; cat. #37-7000); rabbit anti-FBXW11, 1 : 10 000 (Thermo Fisher Scientific, Waltham, MA, USA; cat. #PA5-29878); rabbit anti-hnRNPAB, 1 : 1000 (Santa Cruz, Dallas, TX, USA; cat. #sc-98723); rabbit anti-hnRNPA2B1, 1 : 5000 (Thermo Fisher Scientific; cat. #PA5-30960) and mouse anti-GAPDH, 1 : 5000 (Merck Millipore; cat. #MAB374) antibodies.

### Splicing assay by RT-PCR

RNA purification and reverse transcription were performed with the same method described in quantitative RT-PCR section. Following PCRs were carried out with KOD FX polymerase (TOYOBO) with primers described below: Fly_htt, forward primer: 5′-GTCTCAGCGATAGCGAATCC-3′ and reverse primer: 5′-TATAAGCTGTGGCAGCAGGA-3′. Fly_gapdh, forward primer: 5′-GGTACGACAACGAGTTCGGTTACTC-3′ and reverse primer: 5′-TTTTGGCTAGTTTAGTCCTTGCTCTG-3′. Rat_Htt, forward primer: 5′-CTCATGCAGCCCTGTTCTCT-3′ and reverse primer: 5′- TCAGAAGGAGTCACAGCTGAA-3′. Rat_GAPDH, forward primer: 5-‘TGAACGGGAAGCTCACTGG-3′ and reverse primer: 5-‘TCCACCACCCTGTTGCTGTA-3′. PCR protocol was as follows: Fly: 1 cycle 94 °C for 2 min; 34 cycles 98 °C for 10 s; 34 cycles 58.5 °C for 30 s; 34 cycles 68 °C for 1 min; 1 cycle 12 °C for 5 min followed by cooling to 4 °C. Rat: 1 cycle 94 °C for 2 min; 34 cycles 98 °C for 10 s; 34 cycles 62.9 °C for 30 s; 34 cycles 68 °C for 1 min; 1 cycle 12 °C for 5 min followed by cooling to 4 °C.

### siRNA KD of endogenous hnRNPAB

siRNA for rat hnRNPAB was purchased from Dharmacon (Lafayette, CO, USA; LOC100910156). Three days after plating neurons, the siRNA was transfected using Lipofectamine RNAiMAX (Invitrogen, MA, USA) according to the commercial protocol.

### Plasmid construction

Human hnRNPAB cDNA (858 bp, homo sapiens, hnRNP A/B, NM_004499.3, nt 258-1115) and human hnRNPA2B1 cDNA (1062 bp, homo sapiens, hnRNP A2B1, NM_031243.1, nt 170-1231) were amplified from the RNA of Hela cell using hnRNPAB: forward primer 5′-GGGGAATTCTCGGCCTAGCATGTCGGA-3′ and reverse primer 5′-GGGCTCGAGCATGTGTGCGATCAGTTGGT-3′ hnRNPA2B1: forward primer 5′-GGGGAATTCTCCGCGATGGAGAAAACTTTA-3′ and reverse primer 5′-GGGCTCGAGCCCATGGCAAATAGGAAGAA-3′ containing *Eco*RI or *xho*I site and were subcloned into pCMV-3Tag-1A (Agilent Technologies Inc., Santa Clara, CA, USA).

### Cell death assay

Cell death assays were performed as described previously.^20^ In short, attached cells were directly incubated for 5 min in 0.4% Trypan blue, then blue-stained (nonviable) and non-stained (viable) cells were counted. Six visual fields were randomly selected for each dish.

### Inhibitors

PLK1 inhibitors: BI6727 and ON01910 were obtained from Selleck Chemicals (Selleckchem, Houston, TX, USA; cat. #S2235 and cat. #S1362). Caspase inhibitor: z-VAD-fmk was purchased from Biomol Research Laboratories (Plymouth Meeting, PA, USA; cat. #BML-P416-0001). Necropotosis inhibitor: necrostatin-1 was purchased from Sigma-Aldrich (cat. #N9037).

### *Drosophila* genetics

UAS-YAPins61 transgenic fly was constructed in our lab previously.^20^ The tublin-Gal4/TM3 GFP, Ser was made by crossed w;+/+sb/TM3 GFP, Ser with tublin-Gal4/sb^1^ (BDSC 5138). UAS-yki and sd^1^ flies were obtained from Bloomington Drosophila Stock Center (BDSC). w;+/+sb/TM3 GFP, Ser was a gift from Dr. Sone (Toho University). UAS-dMST^K71R^ (UAS-hpoK71R) was a gift from Dr. Georg Halder.^69^ UAS-dMSTc (UAS-hpoC) was a gift from Dr. Zhi-Chun Lai.^70^

### Microarray analysis

Total RNA was purified from AMA-treated or non-treated wild-type larvae with RNeasy mini kit (Qiagen). To eliminate genomic DNA contamination, on-column DNA digestion was carried out for each sample with DNase I (Qiagen). The purified total RNA samples were performed with Agilent oligo DNA microarray ver.2 (Agilent Technologies Inc.).

## Abbreviations

AMA: *α*-amanitin
CNS: central nervous system
ER: endoplasmic reticulum
FBXW11: F-Box and WD repeat domain containing 11
FTLD: frontotemporal lobar degeneration
FUS: fused in sarcoma/translocated in sarcoma
hnRNA: heterogenous nuclear RNA
hnRNP: heterogeneous nuclear ribonucleoprotein
Htt: huntingtin
KD: knockdown
OE: overexpression
Plk1: polo-like kinase-1
Pol II: RNA polymerase II
RPKM: Reads per kilobase of exon per million mapped fragments
TDP43: TAR DNA-bnding protein 43 kD
TNF: tumor necrosis factor
TRIAD: transcriptional repression-induced atypical cell death of neurons
YAP: yes-associated protein
Yki: yorkie
