# Supplementary figures and images for: The hnRNP-Htt axis regulates necrotic cell death induced by transcriptional repression through impaired RNA splicing
Source: Cell Death Dis. 2016 Apr 28;7(4):e2207–. doi: 10.1038/cddis.2016.101 (PMC4855646; doi:10.1038/cddis.2016.101)

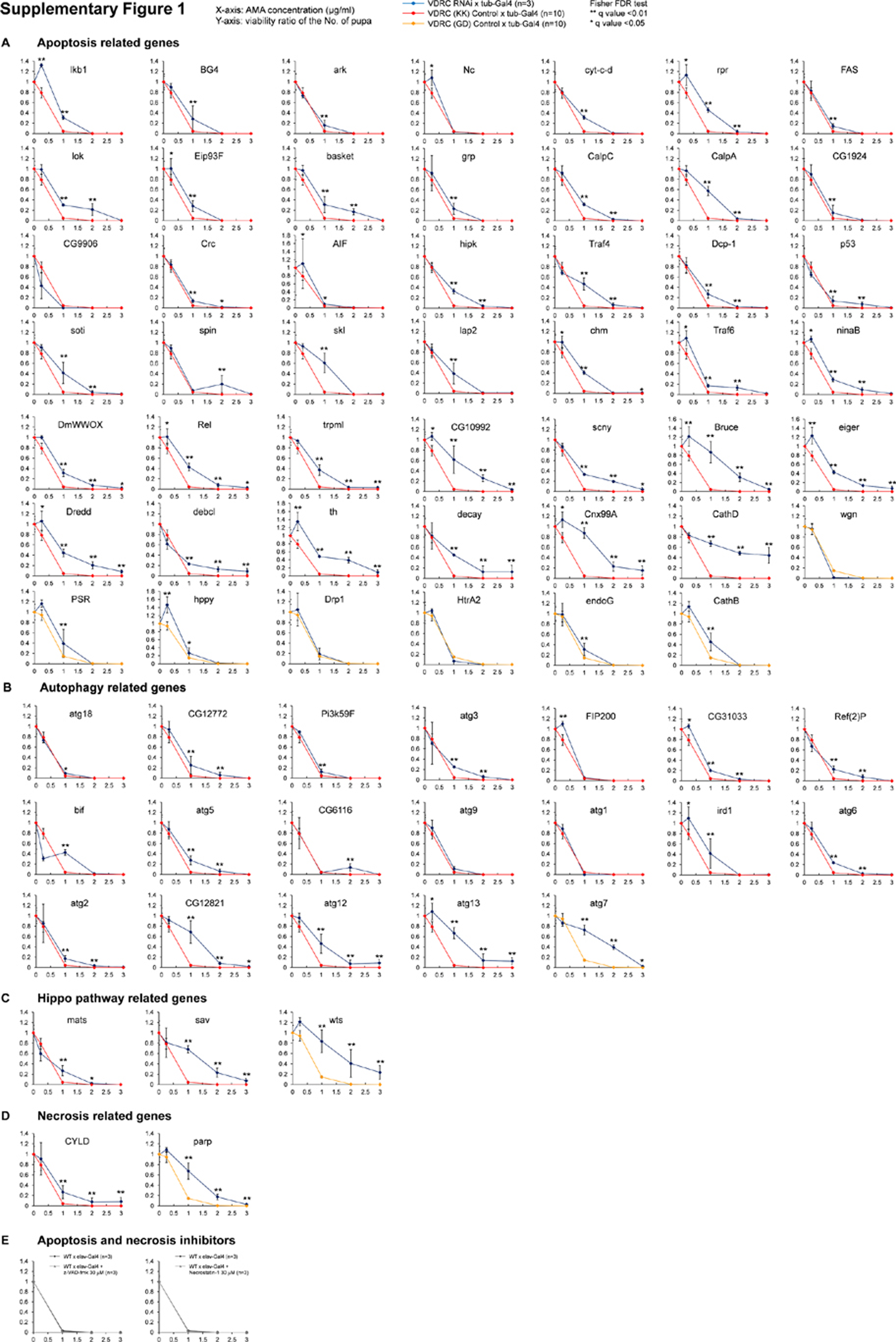

Supplement: Supplementary Figure 1 [file cddis2016101x1.tif]

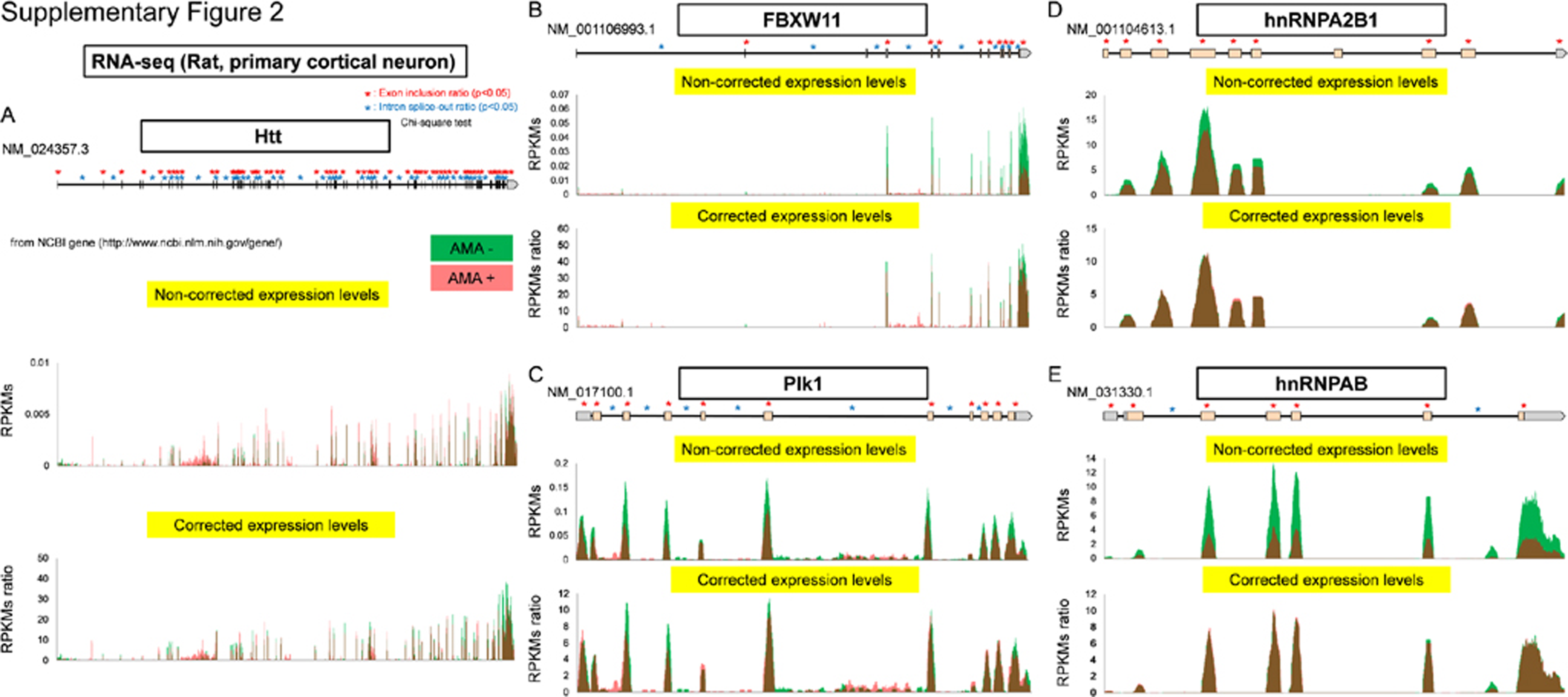

Supplement: Supplementary Figure 2 [file cddis2016101x2.tif]

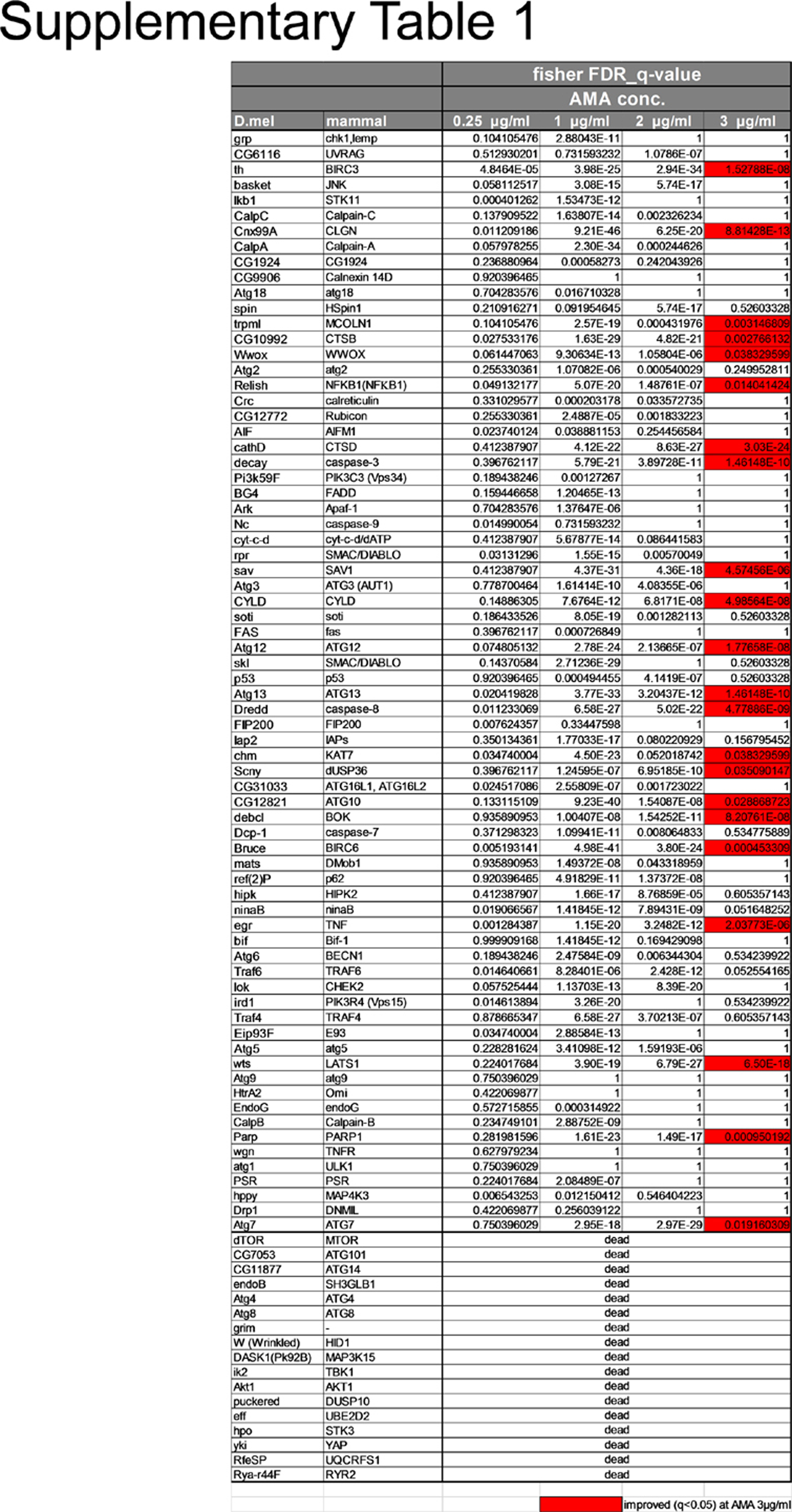

Supplement: Supplementary Table 1 [file cddis2016101x3.tif]
