# Supplementary material for: The hnRNP-Htt axis regulates necrotic cell death induced by transcriptional repression through impaired RNA splicing
Source: Cell Death Dis. 2016 Apr 28;7(4):e2207–. doi: 10.1038/cddis.2016.101 (PMC4855646; doi:10.1038/cddis.2016.101)
Supplement: Supplementary Legends [file cddis2016101x4.doc]

**Supplementary Figure 1**

**Raw data of the KD fly screening**

Raw data of the KD fly screening are shown with categories of their functions described in KEGG, which corresponds to the categories in Figure 2D.Asterisks indicate statistical difference in Fischer FDR test.

**Supplementary Figure 2**

**Impaired RNA splicing in TRIAD of rat cortical neurons**

(A) Transcriptional levels and splicing changes of rat Htt in primary cortical neurons (E17) 24 hours after addition of α-amanitin (AMA) were evaluated by RNA sequencing with a NGS. Non-corrected expression levels represent total expression levels with read per kilobase of exon per million mapped sequence reads (RPKM). Corrected expression levels represent RPKM ratio corrected by

total gene expression levels of Htt in the absence or presence of α-amanitin.

(B) Transcriptional levels and splicing changes of rat FBXW11 were evaluated by RNA sequencing with a NGS.

(C) Transcriptional levels and splicing changes of rat Plk1 were evaluated by RNA sequencing with a NGS.

(D) Transcriptional levels and splicing changes of rat hnRNPA2B1 were evaluated by RNA sequencing with a NGS.

(E) Transcriptional levels and splicing changes of rat hnRNPAB were evaluated by RNA sequencing with a NGS.

**Supplementary Table 1**

Statistical analyses of each gene in the KD screening are shown in the list.
